# Unilateral Optic Disc Edema in a Paediatric Patient: Diagnostic Dilemmas and Management

**DOI:** 10.1155/2010/529081

**Published:** 2010-12-09

**Authors:** Evgenia Kanonidou, Irini Chatziralli, Christina Kanonidou, Maria Parava, Nikolas Ziakas

**Affiliations:** ^1^Department of Ophthalmology, General Hospital of Veria, 97 Vlasi Gavriilidi Street, 55131 Veria, Greece; ^2^Department of Microbiology, AHEPA University Hospital, Aristotle University of Thessaloniki, 54636 Thessaloniki, Greece; ^3^Department of Neurology, Petra-Olympou Psychiatric Hospital, 60100 Katerini, Greece; ^4^Department of Ophthalmology, AHEPA University Hospital, Aristotle University of Thessaloniki, 54636 Thessaloniki, Greece

## Abstract

*Introduction*. We report a case of unilateral optic disc edema in a paediatric patient and discuss the concerns involved in diagnosis and management of similar cases. *Materials and Methods*. Female aged 10 years was referred to our clinic due to progressive visual loss of the LE over a few days. Her visual acuities (VA) were RE 10/10, LE 3/10, and she had a relative afferent pupillary defect and decreased colour vision in her LE and normal and painless eye movements. Fundoscopy showed a remarkably swollen disc of the LE, and visual field (VF) examination revealed enlargement of the blind spot and presence of horizontal inferior papillomacular scotoma. Neurological examination, CT of brain and orbits and blood tests were normal. Visual evoked potentials revealed an obstacle in the myelin substance before the optic chiasma of the LE. *Results*. The patient was treated with intravenous methylprednoslone for 3 days and with oral methylprednizole for 15 days in progressively diminished daily doses. This led to gradual improvement of VA, colour vision, and visual field and resolution of optic disc oedema. *Discussion*. Concerns that have to be taken into account regarding diagnosis and management of similar cases are related to lumbar puncture indications, treatment with corticosteroids, and appropriate followup.

## 1. Introduction

Optic neuritis in childhood is usually attributed to viral causes (66%) and in most cases is bilateral, affecting both eyes simultaneously [1]. As for the therapeutic approach, there are no clinical trials with corticosteroids in children. In both unilateral and bilateral attacks with minor visual loss, a periodical followup is recommended whereas in unilateral and bilateral attacks with medium to severe visual loss pharmaceutical therapy is advised with intravenous methylprednisolone 15 mg/kg/day for 3 days and periodical followup. Per os therapy is not required. However, recent studies compare methylprednisolone IV alone or followed by oral corticosteroids, the second being more effective in preventing relapses [2]. 

Prognosis concerning vision is poor compared with adults, although the risk of developing multiple sclerosis is lower. Children with unilateral optic neuritis present better vision prognosis (100% > 20/40) but develop multiple sclerosis (MS) more often compared to children with bilateral optic neuritis (75%) [1]. However, other studies conclude that bilateral and not unilateral optic neuritis in children precede more often MS [3]. Patients developing MS are older at the onset of optic neuritis (12 years old) compared to those who do not develop MS (9 years old) [1].

We present an interesting case of unilateral optic disc edema in a paediatric patient and discuss the concerns involved in diagnosis and management of similar cases.

## 2. Case Presentation

**Figure 1:**
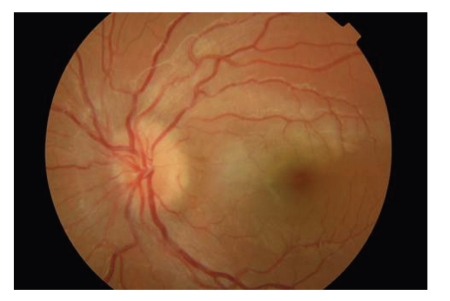
Fundus photography of the patient's LE at the time of admission in which a remarkably swollen optic disc is illustrated.

A 10-year-old female patient was referred to our clinic due to progressive visual loss of the LE described as blurred vision over 3 days without further symptoms. On examination, the visual acuities (VA) were RE 10/10, LE 3/10; she had a relative afferent pupillary defect and decreased colour vision in her LE and normal and painless eye movements. Cycloplegic examination revealed hypermetropia of +0.75 sph in the LE while fundoscopy showed remarkably swollen optic disc (Figure 1). Her past medical history included periodical bronchial asthmatic attacks and pneumonia a year ago, while family history was clear.

**Figure 2:**
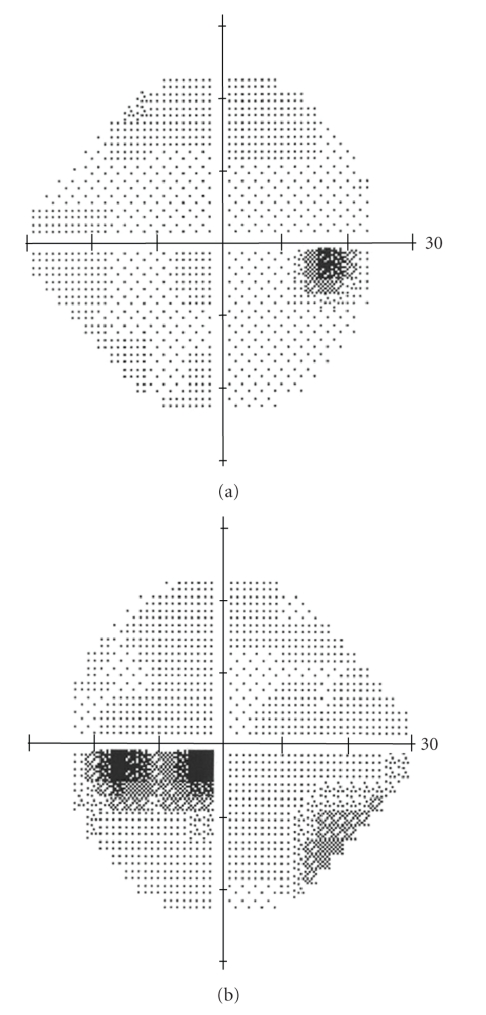
Normal visual field examination in RE (a) Visual field examination with an enlargement of the blind spot and presence of horizontal inferior papillomacular scotoma in LE (b).

Taking the differential diagnosis of optic disc edema into consideration, further investigations were performed. Visual field examination revealed enlargement of the blind spot and presence of horizontal inferior papillomacular scotoma in the LE (Figure 2). Neurological examination was thorough and unremarkable. Computed tomography (CT) and magnetic resonance imaging (MRI) of brain and orbits and all blood tests (full blood count, basic biochemical analysis, CRP, blood coagulation examinations, FT3, FT4, TSH, Anti-TPO, Anti-TG, IgM, IgE, IgG, IgA, RF, ANA, ANCA, and acL) were normal. The existence of adenovirus and RSV antibodies was attributed to cross-reaction or to the use of cortisone and thus was not evaluated. Finally, visual evoked potentials of the occipital lobe revealed prolongation of the P100 latencies after the stimulation of the LE (172.8 ms in LE versus 114.3 ms in RE, at 17′′, and 151.8 ms in LE versus 101.4 ms in RE, at 70′′) indicative of an obstacle in the myelin substance before the optic chiasma.

The patient was treated with intravenous methylprednizolone 500 mg daily for 3 days (Solumedrol 500 mg, Pfizer) and afterwards with oral methylprednizole (Medrol, Pfizer, 1 mg/kgr body weight divided in 2 daily doses) for 15 days in progressively diminished doses. 

**Figure 3:**
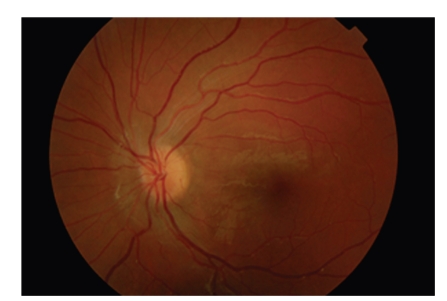
Fundus photography of the patient's LE ten days after being released in which a remarkable improvement of the optic disc edema is observed.

Five days after initiation of treatment (8 days after the onset of symptoms) VA of the LE increased to 7/10, colour vision and pupillary reflexes were recorded as normal, the optic disc swelling improved, and the patient was released. At the followup, ten days later VA of the LE was 10/10, there was a remarkable improvement of the optic disc oedema (Figure 3) and visual field examination was normal. After 20 days the VA remained 10/10 and the optic disc was found absolutely normal in fundoscopy.

Written informed consent was obtained from the patient.

## 3. Discussion

**Figure 4:**
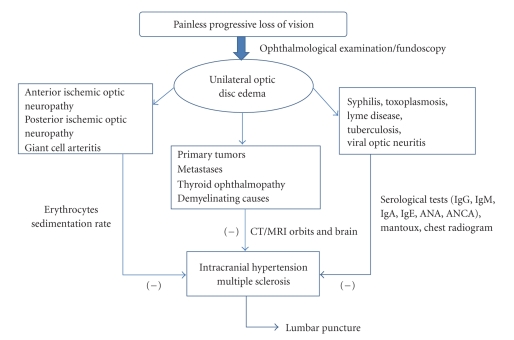
Differential diagnosis of unilateral optic disc oedema and diagnostic approach.

Differential diagnosis of optic disc oedema usually includes intracranial tumours, benign intracranial hypertension, hydrocephalus and optic neuritis due to demyelinating, and viral, infectious, and idiopathic causes. However, it can also appear in ischemic optic neuropathy, neoplasmatic infiltration, sarcoidosis, syphilis, and toxoplasmosis (Figure 4). Furthermore, eye conditions such as uveitis, hypotonia, and central retinal vein thrombosis or systemic diseases such as malignant hypertension, severe anaemia, and hypoxaemia can lead to disc oedema. Finally, compression due to Grave's disease, orbital lesions, and trauma can cause optic disc oedema, as well as the hereditary Leber neuropathy [4]. Many of the situations included in the differential diagnosis are extremely rare in children and seldom present by isolated unilateral optic disc oedema. 

There is still disagreement whether lumbar puncture should be indicated in the differential diagnosis of unilateral optic disc oedema in paediatric patients. In a study of 15 patients with unilateral optic disc oedema, 10 had idiopathic intracranial hypertension. Neuroimaging did not reveal any causes of the oedema, the other optic nerve was normal and the visual disorders were similar to those with typical bilateral oedema due to idiopathic intracranial hypertension [5]. Although unilateral optic disc oedema due to idiopathic intracranial hypertension among paediatric patients is a rare condition, the use of lumbar puncture is not clearly indicated. Nevertheless, despite being an interventional diagnostic technique, lumbar puncture can be useful not only to measure intracranial pressure, but also to analyse cellularity, proteins, IgG production, and specific antibodies including Aquaporin 4 Antibodies (AQP4-Ab), that may be important in defining followup and risks of evolution to multiple sclerosis.

Similar scientific controversy exists regarding the corticosteroid treatment of paediatric patients with unilateral optic disc oedema [6]. It is well known that longstanding untreated disc oedema results in atrophy and permanent vision impairment [4]. Patients treated with steroids only per os show a higher relapse risk [7]. Although patients treated with intravenous corticosteroids show an earlier improvement, the long-term results (6 months-1 year) are equal for both groups. There is also a significant relapse risk when intravenous therapy is not followed by per os treatment. In the 1980s, the optic neuritis treatment trial (ONTT) was developed to evaluate corticosteroid treatment for optic neuritis. This multicenter randomized clinical trial showed that high-dose intravenous methylprednisolone followed by oral prednisone accelerated visual recovery but did not improve the 6-month or 1-year visual outcome compared with placebo, whereas treatment with oral prednisone alone did not improve the outcome and was associated with an increased rate of recurrences of optic neuritis. Another finding was that those who received intravenous corticosteroids followed by oral corticosteroids had a temporarily reduced risk of development of a second demyelinating event consistent with MS [8]. On the other hand, considering the side effects of steroids, it is difficult to conclude in a therapeutic protocol. In any case, it is the clinical doctor who has to evaluate the quality of life, the potential dangers for the patient, and the visual function and to take a decision regarding treatment.

The followup of paediatric patients with unilateral optic disc oedema should include examination of visual acuity, colour vision, and pupillary reflexes. Also visual field examination, visual evoked potentials, and CT/MRI of brain and orbits should be undertaken. The risk of subsequent MS development can now be reliably estimated and MRI is established as the single most important predictor. However, the Optic Neuritis Study Group found that still 25% of optic neuritis with no lesions on brain MRI evolved to MS after an initial episode; therefore a long followup by a paediatric neurologist is strongly recommended [8–10]. 

Concerns that have to be taken into account regarding diagnosis and treatment of similar cases are related to the frequency and type of followup, since in the majority of cases the underlying cause seems to be a viral infection and the risk of developing multiple sclerosis is rather low.

## References

[1] Morales DS, Siatkowski RM, Howard CW, Warman R (2000). Optic neuritis in children. *Journal of Pediatric Ophthalmology and Strabismus*.

[2] Hickman SJ, Dalton CM, Miller DH, Plant GT (2002). Management of acute optic neuritis. *The Lancet*.

[3] Wilejto M, Shroff M, Buncic JR, Kennedy J, Goia C, Banwell B (2006). The clinical features, MRI findings, and outcome of optic neuritis in children. *Neurology*.

[4] Balcer LJ, Beck RW, Yanoff M, Duker’s JS (2004). Inflammatory optic neuropathies and neuroretinitis. *Ophthalmology*.

[5] Huna-Baron R, Landau K, Rosenberg M, Warren FA, Kupersmith MJ (2001). Unilateral swollen disc due to increased intracranial pressure. *Neurology*.

[6] Kaufman DI, Trobe JD, Eggenberger ER, Whitaker JN (2000). Practice parameter: the role of corticosteroids in the management of acute monosymptomatic optic neuritis. report of the Quality Standards Subcommittee of the American Academy of Neurology. *American Journal of Ophthalmology*.

[7] Beck RW, Trobe JD, Moke PS (1997). Visual function 5 years after optic neuritis: experience of the optic neuritis treatment trial. *Archives of Ophthalmology*.

[8] Volpe NJ (2008). The optic neuritis treatment trial: a definitive answer and profound impact with unexpected results. *Archives of Ophthalmology*.

[9] Wilejo M, Shroff M, Buncic JR, Kennedy J, Goia C, Banwell B (2008). Optic neuritis study group. Multiple sclerosis risk after optic neuritis: final optic neuritis treatment follow-up. *Archives of Neurology*.

[10] Absoud M, Cummins C, Desai N Childhood optic neuritis clinical features and outcome.

